# Ubc13 haploinsufficiency protects against age-related insulin resistance and high-fat diet-induced obesity

**DOI:** 10.1038/srep35983

**Published:** 2016-10-31

**Authors:** Erina Joo, Toru Fukushima, Norio Harada, John C. Reed, Shu-ichi Matsuzawa, Nobuya Inagaki

**Affiliations:** 1 Department of Diabetes, Endocrinology and Nutrition, Graduate School of Medicine, Kyoto University, 54 Kawahara-cho, Shogoin, Sakyo-ku, Kyoto 606-8507, Japan; 2 Sanford-Burnham-Prebys Medical Discovery Institute, 10901 North Torrey Pines Road, La Jolla, CA 92037, USA; 3 Roche, Pharma Research & Early Development, Grenzacherstrasse 124, 4070 Basel, Switzerland; 4 Department of Neurology, Graduate School of Medicine, Kyoto University, 54 Kawahara-cho, Shogoin, Sakyo-ku, Kyoto 606-8507, Japan

## Abstract

Obesity is associated with low-grade inflammation that leads to insulin resistance and type 2 diabetes via Toll-like Receptor (TLR) and TNF-family cytokine receptor (TNFR) signaling pathways. Ubc13 is an ubiquitin-conjugating enzyme responsible for non-canonical K63-linked polyubiquitination of TNF receptor-associated factor (TRAF)-family adapter proteins involved in TLR and TNFR pathways. However, the relationship between Ubc13 and metabolic disease remains unclear. In this study, we investigated the role of Ubc13 in insulin resistance and high-fat diet (HFD)-induced obesity. We compared wild-type (WT) and Ubc13 haploinsufficient (*ubc13*^+/−^) mice under normal diet (ND) and HFD, since homozygous knockout mice (*ubc13*^−/−^) are embryonic lethal. Male and female *ubc13*^+/−^ mice were protected against age-related insulin resistance under ND and HFD compared to WT mice. Interestingly, only female *ubc13*^+/−^ mice were protected against HFD-induced obesity and hepatic steatosis. Moreover, only female HFD-fed *ubc13*^+/−^ mice showed lower expression of inflammatory cytokines that was secondary to reduction in weight gain not present in the other groups. In summary, our results indicate that suppression of Ubc13 activity may play a metabolic role independent of its inflammatory function. Thus, Ubc13 could represent a therapeutic target for insulin resistance, diet-induced obesity, and associated metabolic dysfunctions.

Obesity is now reaching pandemic proportions worldwide^1^ and is closely associated with chronic low-grade inflammation, which causes insulin resistance and other metabolic diseases such as hypertension and atherosclerosis^2^,^3^. Recent studies suggest that obesity induces immune cell infiltration and activation in liver and adipose tissue accompanied by secretion of inflammatory cytokines and chemokines, adversely affecting insulin sensitivity^4^. The link between obesity and insulin resistance is disrupted by gene knockout of mediators of inflammatory pathways such as Toll-like Receptor (TLR)^5^,^6^,^7^,^8^, TNF-family cytokine receptor (TNFR)^9^, and nucleotide-binding domain, leucine-rich repeats containing family, pyrin domain-containing-3 (Nlrp3) inflammasome^10^. In addition, aging is associated with chronic low-grade inflammation. Recently, mice lacking Nlrp3 were reported to show improved age-related disorders including type 2 diabetes^11^, suggesting that suppression of excessive inflammatory action induced by obesity and aging might improve these associated metabolic diseases.

Ubc13 is required for signaling by a variety of receptors important in immune regulation. Ubc13 is a non-canonical ubiquitin-conjugating enzyme (E2) that catalyzes formation of lysine 63 (K63)-linked polyubiquitin chains on various target proteins^12^,^13^. Unlike the canonical K48-linked polyubiquitin chains, K63-linked polyubiquitin chains are not substrates for the proteasome^14^,^15^. Instead, K63-linked polyubiquitination plays essential roles in protein activation and interaction^16^,^17^,^18^. Ubc13 binds the RING domains of TRAF-family adapter proteins such as TRAF2 and TRAF6, and promotes activation of protein kinases involved in signaling by TNFRs^19^,^20^ and by TLRs^21^. With regard to the role of Ubc13 in TRAF-mediated signaling, lipopolysaccharide (LPS) and cytokines such as TNFα and IL-1β that activate TRAF2 or TRAF6 result in its non-canonical ubiquitination and subsequent downstream activation of kinases, including JNK. Ubc13 is also associated with the activation of NF-κB, via its effect on TRAF6, resulting in activation of the kinase TAK1^17^.

Previously, we have reported *in vivo* functions of Ubc13 in the context of innate immune responses, using the method of targeted gene ablation in mice^22^. We reported that Ubc13 homozygous knockout mice were embryonic lethal and that heterozygous *ubc13*^+/−^ mice have normal phenotypes, despite reduced levels of Ubc13 protein. However, *ubc13*^+/−^ mice exhibit marked reductions in responsiveness to challenge with LPS^22^. Moreover, *ubc13*^+/−^ splenocytes and bone marrow-derived macrophages (BMDMs) display blunted responses to LPS and TNF with respect to cytokine secretion and various TNFR/TLR-mediated signal transduction events^22^. Although Ubc13 was reported to play a critical role *in vivo* in inflammatory responses, its role in Ubc13 regulation of chronic low-grade inflammation and insulin resistance is not known.

In the present study, we investigated the role of Ubc13 in insulin resistance and HFD-induced obesity using *ubc13*^+/−^ mice. We found that insulin resistance was improved in male HFD-fed and male and female ND-fed aged mice without any change in inflammatory status or weight gain. Interestingly, female *ubc13*^+/−^ mice are protected against HFD-induced obesity, hepatic steatosis, and insulin resistance. Ubc13 haploinsufficiency only had an effect on inflammation in female mice on HFD that was secondary to reduction in weight gain not present in the other groups. Therefore, this study suggests that Ubc13 haploinsufficiency has a metabolic role that is independent of its function in inflammatory signal transduction.

## Results

### Effect of Ubc13 haploinsufficiency on body composition, food intake, and energy expenditure

**Figure 1:**
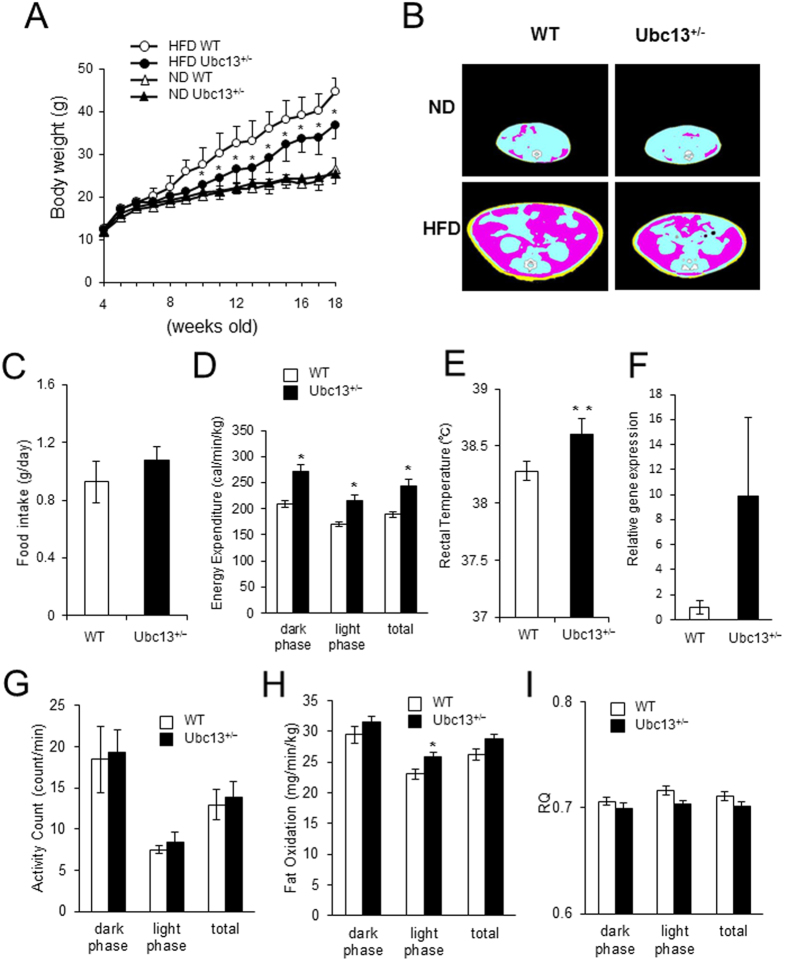
Female *ubc13*^+/−^ mice display decreased weight gain and increased energy expenditure on HFD. (**A**) Body weights of female WT versus *ubc13*^+/−^ mice on ND or HFD (n = 5 to 6). HFD was started at 4 weeks of age. *p < 0.05 versus HFD-fed WT mice. (**B**) Representative CT scanning images of abdominal cross-sections of WT and *ubc13*^+/−^ mice on ND or HFD. Pink, yellow, and blue areas represent visceral fat, subcutaneous fat, and lean mass, respectively. (**C**–**E**) Food intake (**C**), energy expenditure (**D**), and rectal temperature (**E**) were measured for female WT and *ubc13*^+/−^ mice on a HFD (n = 4 to 6). *p < 0.05, **p < 0.01 versus HFD-fed WT mice. (**F**) qRT-PCR analysis of the expression of the gene encoding *UCP1* in VAT of female WT and *ubc13*^+/−^ mice on HFD (n = 5 to 6). (**G**–**I**) activity count (**G**), fat oxidation (**H**), and RQ (**I**) were measured for female WT and *ubc13*^+/−^ mice on a HFD (n = 4). *p < 0.05, versus HFD-fed WT mice.

To investigate the functional role of Ubc13 in HFD-induced obesity and insulin resistance, we fed 4-week-old *ubc13*^+/+^ (WT) and *ubc13*^+/−^ mice either ND or HFD for 14 weeks. On ND, no statistical differences were observed in body weight, food intake, and energy expenditure between WT and *ubc13*^+/−^ mice (Fig. 1A,B, Figs S1 and S2A,B). In contrast, on HFD, male *ubc13*^+/−^ mice tended to exhibit lower body weight than WT mice, whereas female *ubc13*^+/−^ mice exhibited significantly lower body weight than WT mice (Fig. 1A and Fig. S1). CT scan imaging revealed decreases in both visceral and subcutaneous adipose tissues of female *ubc13*^+/−^ mice on HFD (Fig. 1B). Food intake and locomotor activity were similar in female WT and *ubc13*^+/−^ mice (Fig. 1C,G), whereas energy expenditure, fat oxidation, and rectal temperature were significantly increased in female *ubc13*^+/−^ mice compared to WT mice on HFD (Fig. 1D,E,H). In addition, *uncoupling protein 1*(*UCP1*) expression in visceral adipose tissue (VAT) tended to be increased and respiratory quotient (RQ) tended to be decreased in HFD-fed female *ubc13*^+/−^ mice compared to WT mice (Fig. 1F,I).

### Effect of Ubc13 haploinsufficiency on insulin resistance under ND and HFD

**Figure 2:**
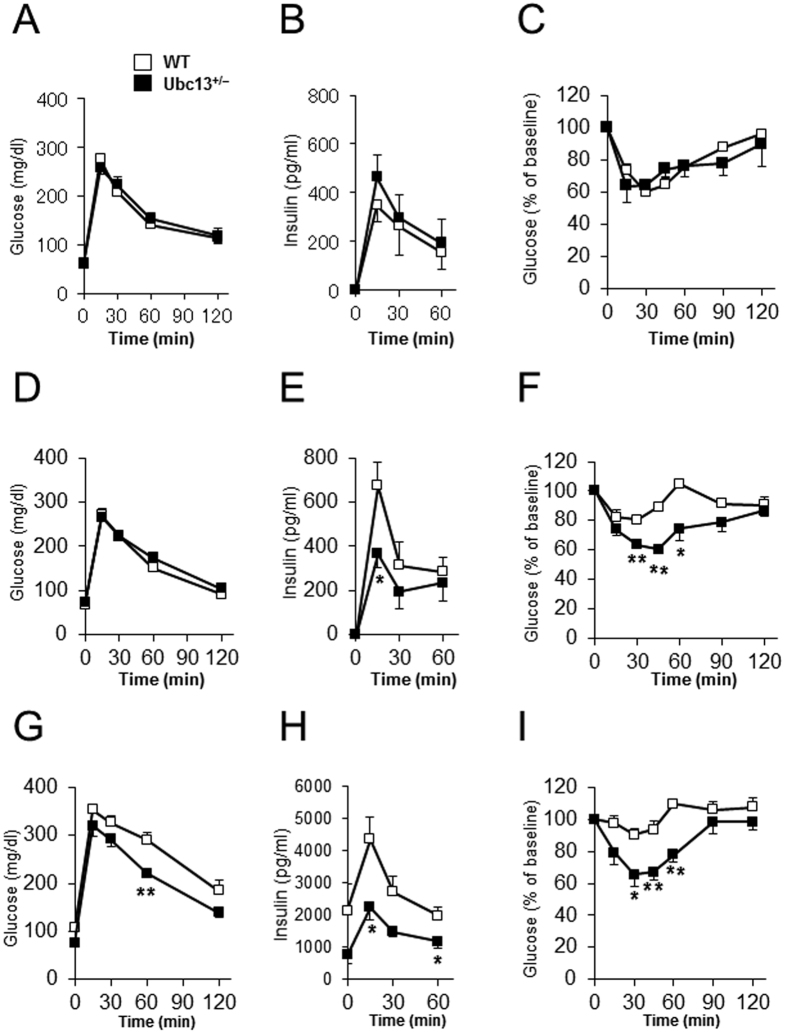
Female *ubc13*^+/−^ mice are protected against age-related and HFD-induced insulin resistance. (**A**–**C**) Blood glucose (**A**) and serum insulin (**B**) levels during OGTT and blood glucose (**C**) levels during ITT were measured for female WT and *ubc13*^+/−^ mice at 8 weeks of age on ND (n = 4). (**D**–**F**) Blood glucose (**D**) and serum insulin (**E**) levels during OGTT and blood glucose (**F**) levels during ITT were measured for female WT and *ubc13*^+/−^ mice at 18 weeks of age on ND (n = 6). *p < 0.05, **p < 0.01 versus WT mice. (**G**–**I**) Blood glucose (**G**) and serum insulin (**H**) levels during OGTT and blood glucose (**I**) levels during ITT were measured for female WT and *ubc13*^+/−^ mice after 14 weeks of HFD (n = 5 to 6). HFD was started at 4 weeks of age. *p < 0.05, **p < 0.01 versus HFD-fed WT mice.

We also evaluated the impact of Ubc13 haploinsufficiency on insulin sensitivity using oral glucose tolerance test (OGTT) and insulin tolerance test (ITT). No statistical differences in OGTT and ITT data were observed between 8-week-old WT and *ubc13*^+/−^ mice after 4 weeks of ND (Fig. 2A–C). Interestingly, plasma insulin levels during OGTT were lower in 18-week-old female *ubc13*^+/−^ mice versus WT mice despite a lack of difference in plasma glucose levels after 14 weeks of ND (Fig. 2D,E). Consistent with these results, insulin sensitivity was significantly enhanced in 18-week-old female *ubc13*^+/−^ mice during ITT (Fig. 2F). On HFD, the mice became hyperinsulinemic; however, plasma insulin levels during OGTT and plasma glucose levels during OGTT and ITT were lower in 18-week-old female *ubc13*^+/−^ mice compared to WT mice after 14 weeks of HFD (Fig. 2G–I). In male mice, plasma glucose and insulin levels during OGTT were also significantly lower in 18-week-old *ubc13*^+/−^ mice versus WT mice after 14 weeks of ND (Fig. S3A,B). However, plasma glucose levels during ITT tended to be decreased in 18-week-old *ubc13*^+/−^ mice (Fig. S3C). Moreover, male HFD-fed *ubc13*^+/−^ mice tended to show improved insulin resistance compared to WT mice after 14 weeks of HFD (Fig. S3D–F), indicating that the effects of Ubc13 haploinsufficiency were mild in male mice compared to female mice. Although the reason for these sex differences is currently unclear, our results demonstrate that Ubc13 haploinsufficiency can ameliorate age-related and HFD-induced insulin resistance.

### Female *ubc13* ^+/−^ mice are protected against HFD-induced hepatic steatosis and insulin resistance in liver and skeletal muscle

**Figure 3:**
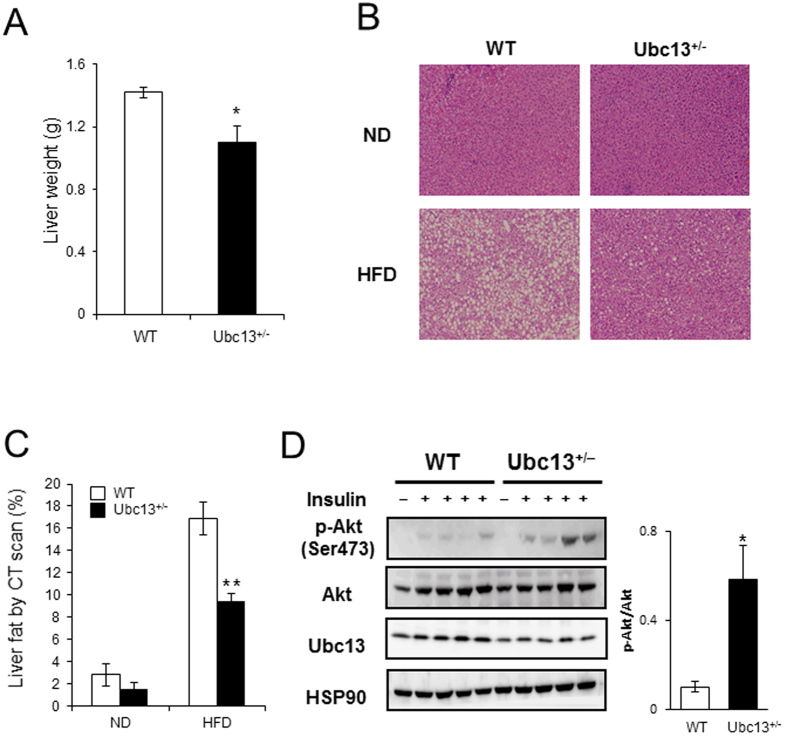
Female *ubc13*^+/−^ mice are protected against HFD-induced hepatic steatosis and insulin resistance. (**A**) Liver weights of female WT and *ubc13*^+/−^ mice were measured after 14 weeks of HFD (n = 5 to 6). *p < 0.05 versus HFD-fed WT mice. (**B**) Representative photographs of H&E staining of liver from female WT and *ubc13*^+/−^ mice on ND or HFD. (**C**) Liver densities of female WT and *ubc13*^+/−^ mice were calculated from abdominal CT scan on ND or HFD (n = 5 to 6). **p < 0.01 versus HFD-fed WT mice. (**D**) Liver lysates from female WT and *ubc13*^+/−^ mice on HFD were analyzed by immunoblotting using antibodies for p-Akt (Ser473), total Akt, and Ubc13. The membrane was reprobed with anti-HSP90 antibody as a control. The intensities of phosphorylated-Akt after insulin injection were normalized to total Akt protein levels.

As chronic exposure of mice to HFD causes increased body weight and hepatic lipid accumulation related to insulin resistance^4^, we examined the status of the liver in female WT and *ubc13*^+/−^ mice. On ND, no significant differences were observed in liver weight and fat accumulation between 18-week-old female WT and *ubc13*^+/−^ mice (Fig. S4A,B). On HFD, liver weight and lipid accumulation were reduced in 18-week-old female *ubc13*^+/−^ mice compared to WT mice (Fig. 3A,B). Measuring liver density by CT scan imaging confirmed clear decreases in lipid accumulation in female *ubc13*^+/−^ mice on HFD (Fig. 3C).

We then examined tissue-specific insulin sensitivity by immunoblotting. Akt phosphorylation was enhanced in livers of female HFD-fed *ubc13*^+/−^ mice after insulin stimulation (Fig. 3D). Although there was no histological difference in muscle between 18-week-old female WT and *ubc13*^+/−^ mice on a HFD, Akt phosphorylation was enhanced in muscles of HFD-fed *ubc13*^+/−^ mice after insulin stimulation (Fig. S5A,B). These results support the interpretation that Ubc13 haploinsufficiency increases insulin sensitivity in female *ubc13*^+/−^ mice.

### Female *ubc13* ^+/−^ mice are protected against HFD-induced adipose tissue inflammation

**Figure 4:**
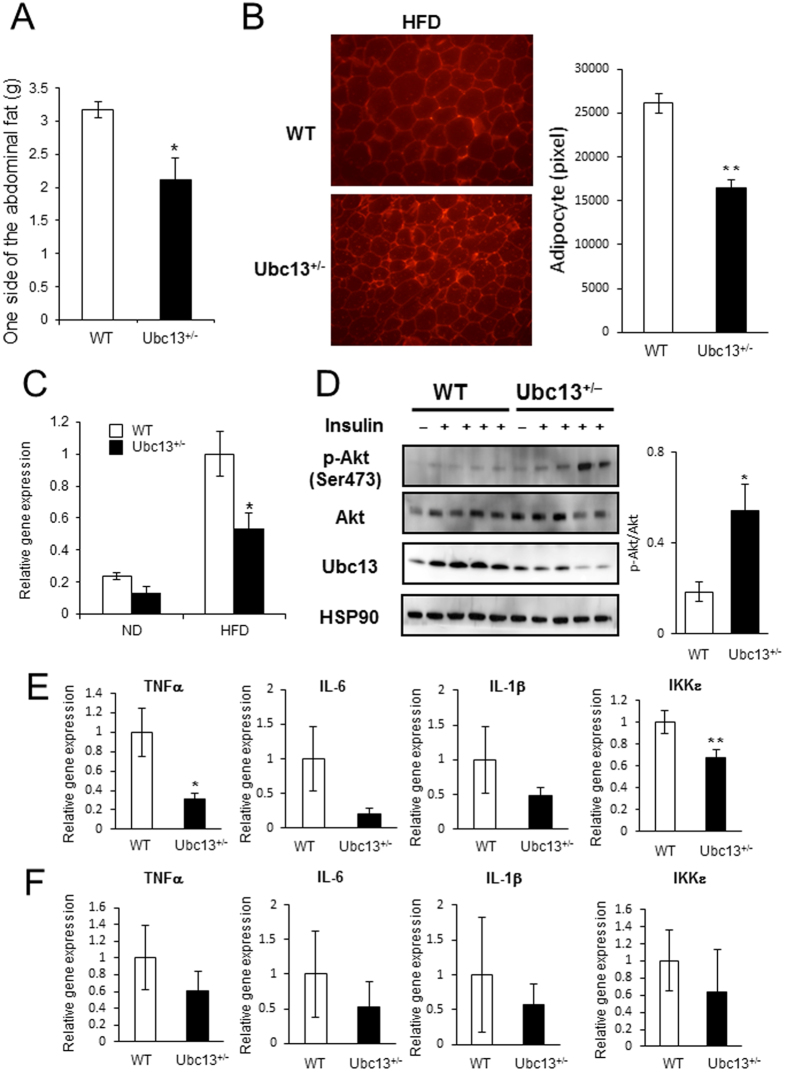
Female *ubc13*^+/−^ mice are protected against HFD-induced adipose tissue inflammation. (**A**) VAT weights of female WT and *ubc13*^+/−^ mice were measured after 14 weeks of HFD (n = 5 to 6). *p < 0.05 versus HFD-fed WT mice. (**B**) Immunohistochemical analysis of perilipin-2 in VAT from female WT and ubc13^+/−^ mice on HFD. Adipocyte size of female WT and ubc13^+/−^ mice on the HFD (n = 5). **p < 0.01 versus HFD-fed WT mice. (**C**) qRT-PCR analysis on the expression of the gene encoding F4/80 in VAT of female WT and *ubc13*^+/−^ mice on HFD (n = 5 to 6). *p < 0.05 versus HFD-fed WT mice. (**D**) VAT lysates from female WT and *ubc13*^+/−^ mice on HFD were analyzed by immunoblotting using antibodies for p-Akt (Ser473), total Akt, and Ubc13. The membrane was reprobed with anti-HSP90 antibody as a control. The intensities of phosphorylated-Akt after insulin injection were normalized to total Akt protein levels. (**E**,**F**) qRT-PCR analysis of the expression of the gene encoding *TNFα*, *IL-6*, *IL-1β*, and *IKKε* in VAT of female WT and *ubc13*^+/−^ mice on HFD (n = 5 to 6) (**E**) and ND (n = 6) (**F**). *p < 0.05, **p < 0.01 versus HFD-fed WT mice.

Recent studies have demonstrated that HFD and aging induce chronic low-grade inflammation in adipose tissue, thereby contributing to insulin resistance^4^,^11^. We examined the status of the adipose tissues in female WT and *ubc13*^+/−^ mice. No significant difference was observed in VAT weight on ND, whereas VAT weight was significantly reduced in 18-week-old HFD-fed female *ubc13*^+/−^ mice compared to WT mice (Fig. 4A and S4B). Histological analysis of VAT revealed that adipocyte cell size was smaller in female HFD-fed *ubc13*^+/−^ mice compared to WT mice (Fig. 4B). Because adipose tissue macrophage (ATM) infiltration is closely related to increased VAT weight^4^, we measured gene expression of *F4/80*, a macrophage specific marker, in VAT from WT and *ubc13*^+/−^ mice by qRT-PCR. On ND, F4/80 expression in VAT tended to be reduced in 18-week-old female ubc13^+/−^ mice compared to WT mice. On HFD, *F4/80* expression in VAT was significantly reduced in 18-week-old female *ubc13*^+/−^ mice compared to WT mice (Fig. 4C). ATM is an important source of inflammatory cytokines leading to insulin resistance. We therefore compared insulin sensitivity and cytokine gene expression in 18-week-old HFD-fed female WT and *ubc13*^+/−^ VAT. Akt phosphorylation was enhanced in VAT of female HFD-fed *ubc13*^+/−^ mice after insulin stimulation (Fig. 4D). Moreover, *TNFα* and *IKKε* expression were significantly reduced while *IL-6* and *IL-1β* expression tended to be reduced in VAT of female HFD-fed *ubc13*^+/−^ mice (Fig. 4E). In contrast on ND, there was no difference in *TNFα*, *IL-6*, *IL-1β*, and *IKKε* expression in VAT of *ubc13*^+/−^ mice (Fig. 4F). Furthermore, no significant differences in *F4/80* and inflammatory cytokine expression were observed between male HFD-fed WT and *ubc13*^+/−^ mice despite improved insulin sensitivity (Fig. S6). These results indicate that Ubc13 haploinsufficiency does not directly regulate adipose tissue inflammation but rather the reduction in HFD-induced adipose tissue inflammation in female *ubc13*^+/−^ mice is likely secondary to the reduction in weight gain and adipose mass.

## Discussion

Recent studies have reported that obesity and aging can generate a state of chronic low-grade inflammation, leading to insulin resistance predominantly through NF-κB activation mediated by inflammatory signaling pathways such as TNFR, TLR, and Nlrp3 inflammasome^5^,^9^,^11^. Furthermore, Ubc13 is known to play a critical role in TNFR, TLR, and Nlrp3 signaling pathways. In this study, we found that Ubc13 haploinsufficiency improved insulin sensitivity in matured mice under ND. Additionally, female *ubc13*^+/−^ mice showed increased energy expenditure and improved HFD-induced obesity, hepatic steatosis, and insulin resistance. Recently, it was reported that HFD-fed mice lacking *IKKε*, a direct transcriptional target of NF-κB, showed similar phenotypes to *ubc13*^+/−^ mice due to increased energy expenditure^23^,^24^. In these mice, increased energy expenditure with increased *UCP1* expression but unaccompanied by changes in RQ were observed. On the other hand, our data showed that fat oxidation was significantly increased in HFD-fed female *ubc13*^+/−^ mice compared to WT mice. The mechanism underlying this discrepancy is unknown, but in our study, expression of NF-κB-inducible genes including *IKKε* was found to be reduced in HFD-fed female *ubc13*^+/−^ mice. In addition, *UCP1* expression in VAT tended to be increased in HFD fed female *ubc13*^+/−^ mice compared to WT mice, suggesting that decreased *IKKε* expression may be one of the molecular mechanisms underlying these phenotypes.

Interestingly, even in ND, 18-week-old, but not 8-week-old, *ubc13*^+/−^ mice showed improved insulin sensitivity compared to that in WT mice. The precise mechanism of these age-related effects remains unknown. We previously reported that macrophages and splenocytes derived from ND-fed *ubc13*^+/−^ mice display blunted responses with respect to LPS and TNF-induced cytokine secretion and activation of JNK and NF-κB^22^. These findings suggest that mild suppression of age-related chronic low-grade JNK and NF-κB activation by Ubc13 haploinsufficiency could be effective to improve insulin sensitivity. However, no significant differences were observed in inflammatory cytokine expression between ND fed WT and *ubc13*^+/−^ mice. These results suggest that other unknown functions might also exist between Ubc13 and insulin sensitivity. Recently, it was reported that Endoplasmic Reticulum (ER) stress is closely associated with insulin resistance via TRAF2/JNK activation, which cause metabolic diseases^25^,^26^,^27^,^28^. Since TRAF2 is one of the substrates of Ubc13, ER stress-mediated insulin resistance might be reduced in *ubc13*^+/−^ mice. Further investigation will be required to identify the detailed mechanisms.

The sex difference that we observed in our study is also interesting. Although both male and female *ubc13*^+/−^ macrophages and splenocytes had reduced LPS and TNF-induced signal activation^22^, male *ubc13*^+/−^ mice showed milder phenotypes than female *ubc13*^+/−^ mice compared to those in WT mice. Previously, Shi *et al*. reported that female, not male, mice lacking *TLR4* were protected against HFD-induced insulin resistance through an unknown mechanism^5^. The explanation for the sex difference in our mice is unknown, but the TLR4/Ubc13 signaling pathway could play an important role in the molecular mechanisms *in vivo*. Recently, Zhang *et al*. reported that STAT3 restrains RANK- and TLR4-mediated signaling by suppressing expression of Ubc13^29^. Because estrogen is known to increase STAT3 activation^30^, the effect of estrogen-mediated STAT3 in Ubc13 may play a partial role in the sex difference. Further investigations are required to determine the relationship between STAT3 and Ubc13.

Since Ubc13 is a unique E2 that catalyzes formation of K63-linked polyubiquitin chains on various substrates, Ubc13 inhibitor is expected to be a novel candidate for the treatment of cancer and inflammatory diseases^31^. However, homozygous disruption of *ubc13* resulted in embryonic lethality at a very early stage of development^22^. Various tissue-specific Ubc13-deficient mice have been reported and some of these mice show severe phenotypes^32^,^33^,^34^,^35^. For instance, keratinocyte-specific *ubc13*^−/−^ mice die by postnatal day 2^34^ and Treg cell-specific *ubc13*^−/−^ mice develop autoimmune diseases^35^, indicating that even tissue-specific complete disruption of *ubc13* can lead to severe toxicity with systemic ramifications. However, the haploinsufficient *ubc13*^+/−^ mice used here grow normally and are protected against acute inflammation, suggesting that moderate Ubc13 inhibition may be effective in suppression of excessive inflammation or unknown dysfunction induced by aging and obesity. It remains to be determined whether hypothetical pharmacological inhibitors of Ubc13 could be dosed in a manner that generates an adequate therapeutic index.

In conclusion, we have shown that Ubc13 haploinsufficiency protects against age-related insulin resistance and HFD-induced obesity. Although other functions of Ubc13 still remain unclear and further investigations are required, Ubc13 may have novel potential as a future target for discovering treatments for insulin resistance, obesity, and type 2 diabetes.

## Methods

### Animals

*Ubc13* heterozygous mice were generated as previously described^22^. The mice were backcrossed at least 6 generations onto a C57BL/6 background, and housed in a specific pathogen-free environment at ambient temperature of 25 °C with a dark-light cycle of 10 and 14 hrs, respectively. WT littermates were used as controls. Animal care and procedures were approved by the Animal Care Committee of Kyoto University. The methods were carried out in accordance with the Animal Care Committee of Kyoto University. The mice were weaned at 4 weeks of age, and fed control fat chow (ND; 10% fat, 20% protein, and 70% carbohydrate by energy) or high fat chow (HFD; 60% fat, 20% protein, and 20% carbohydrate by energy) (Research Diets Inc., New Brunswick, NJ) for 14 weeks.

### Oral glucose tolerance test (OGTT)

After a 16 hrs fasting period, OGTTs (0.75 g/kg body weight) were performed. Blood samples were taken at the indicated times (0, 15, 30, 60, and 120 min after glucose loading), and blood glucose levels and plasma insulin levels were measured. Blood glucose levels were determined by the enzyme-electrode method. Plasma insulin levels were determined using enzyme immunoassay (Shibayagi, Gumma, Japan).

### Insulin tolerance test (ITT)

At a dose of 0.4 U/kg body weight (for ND fed mice) or 1 U/kg body weight (for HFD fed mice) human insulin (Novonordisk, Copenhagen, Denmark) was injected subcutaneously after a 2-h fasting period. Blood samples were collected at the indicated times (0, 15, 30, 60, 90, and 120 min after the loading). Blood-glucose levels were measured as described above.

### Energy expenditure

Energy expenditure was evaluated by measuring respiratory quotient and oxygen consumption by indirect calorimetry every 5 min for 24 hrs under the fed condition^36^. Air from the room was pumped through the chamber, and expired gas was dried in a cotton thin column and subjected to gas analysis (Alco System model 2000, Chiba, Japan). Oxygen consumption (VO_2_) and carbon dioxide production (VCO_2_) were measured, and respiratory quotient (RQ), energy expenditure, and fat oxidation were calculated as follows:


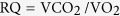


Energy expenditure = 3.816 × VO_2_ + 1.231 × VCO_2_ [cal/min] (by using the Lusk equation^37^).

Fat oxidation = 1.67 × (VO_2_-VCO_2_) [mg/min] (by using Frayn equation^38^).

The locomotor activity of the mice was measured using an automated activity counter (NSAS01; Neuroscience, Tokyo, Japan).

### Computed tomography

Computed tomography (CT) scanning was performed as previously described^39^. Briefly, mice were anesthetized with Nembutal and fixed in a chamber, and transaxially scanned using Latheta (LCT-100M) experimental animal CT system (Aloka, Tokyo, Japan). The whole body was scanned, and contiguous 1-mm slice images of the trunk were used for quantitative assessment (Latheta software, version 1.00). Fat content in liver was quantitatively evaluated.

### Real-time PCR analysis

Total RNA from adipose tissue was extracted using TRIzol solution (Invitrogen, Grand Island, NY, USA) according to the manufacturer’s instructions^40^. For complementary DNA synthesis, 1 μg of total RNA was reverse-transcribed using the reverse transcription system (PrimeScript RT reagent kit, Takara Bio, Shiga, Japan). SYBER Green PCR Master Mix (Applied Biosystems, Foster City, CA, USA) was prepared for real-time quantitative PCR using ABI StepOnePlus Real-Time PCR Systems (Applied Biosystems, Foster City, CA, USA). The PCR was performed for 10 min at 90 °C, followed by 50 cycles at 95 °C for 15 sec and at 60 °C for 1 min. The signal of the products was standardized against the *GAPDH* signal for each sample. Primer pairs for *F4/80*, *TNFα*, *IL-6*, *IL-1β*, *IKKε*, *UCP1*, and *GAPDH* are shown as follows.: *F4/80* (165 bp): 5′-ctt tgg cta tgg gct tcc agt c-3′ and 5′-gca agg agg aca gag ttt atc gtg-3′; *TNFα* (146 bp): 5′-aaa tgg gct ttc cga att ca-3′ and 5′-cag gga aga atc tgg aaa ggt-3′; *IL-6* (78 bp): 5′-gct acc aaa ctg gat ata atc agg a-3′ and 5′-cca ggt agc tat ggt act cca gaa-3′; *IL-1β* (89 bp): 5′-gca act gtt cct gaa ctc aac t-3′ and 5′-atc ttt tgg ggt ccg tca act-3′; *IKKε* (148 bp): 5′-aca agg ccc gaa aca aga aat-3′ and 5′-act gcg aat agc ttc acg atg-3′; *UCP1* (107 bp): 5′-gga ttg gcc tct acg act ca-3′ and 5′-tgc cac acc tcc agt cat ta-3′; *GAPDH* (100 bp): 5′-aaa tgg tga agg tcg gtg tg-3′ and 5′-tcg ttg atg gca aca atc tc-3′.

### Immunohistochemistry

Liver and visceral fat samples were fixed in 10% formalin buffer, embedded in paraffin, and sectioned at 3 μm. The paraffin sections of the liver were stained with hematoxylin and eosin (HE). Images were taken using a microscope with the BZ-8100 system (KEYENCE Corp., Osaka, Japan). The sections of visceral fat were blocked with 3% bovine serum albumin and then incubated overnight at 4 °C with a monoclonal rabbit anti-perilipin 2 antibody (LSBio, Seattle, WA) and afterward with a secondary antibody at room temperature at 1 h. After immunostaining, the mean adipocyte size (surface areas of 15 representative adipocytes per mouse) was analyzed by BZ Analyzer software (KEYENCE Corp.).

### Immunoblotting and antibodies

To compare Akt-phosphorylation levels, insulin (2 U per kg body weight) was injected via inferior vena cava under regular fed condition. 3 min after the injection, adipose tissue, liver tissue, and muscles were collected. Frozen tissue extracts were minced and homogenized in cold RIPA buffer containing 50 mM Tris-HCl (pH 7.4), 150 mM NaCl, 1% NP40, 0.1% SDS, 1 mM EDTA, phosphatase inhibitor cocktail (Sigma-Aldrich), and protease inhibitor cocktail (Roche), and then were centrifuged at 12,000 × g for 5 min. Equal amounts of cell lysates were subjected to immunoblot analysis. Anti-Akt, anti-phospho-Akt (Ser473) antibodies were obtained from Cell Signaling. Quantification of bands on western blot was accomplished by scanning the blots, then determining the densities of the bands using ImageJ software (National Institutes of Health, Bethesda, MD, http://imagej.nih.gov/ij/, 1997–2012). Anti-Ubc13 was obtained from Zymed laboratories. Anti-HSP90 antibody was purchased from Santa Cruz Biotechnology.

### Statistical analysis

All data were expressed as mean ± SE. Statistical analysis was performed using Student’s *t*-test. Significant difference was considered to be present at *p* < 0.05.

## Additional Information

**How to cite this article**: Joo, E. *et al*. Ubc13 haploinsufficiency protects against age-related insulin resistance and high-fat diet-induced obesity. *Sci. Rep.* **6**, 35983; doi: 10.1038/srep35983 (2016).

**Publisher’s note:** Springer Nature remains neutral with regard to jurisdictional claims in published maps and institutional affiliations.

## Supplementary Material

Supplementary Information

## References

[b1] Hossain P., Kawar B. & El Nahas M. Obesity and diabetes in the developing world–a growing challenge. The New England journal of medicine 356, 213–215, 10.1056/NEJMp068177 (2007). 17229948

[b2] Hotamisligil G. S. Inflammation and metabolic disorders. Nature 444, 860–867, 10.1038/nature05485 (2006). 17167474

[b3] Schenk S., Saberi M. & Olefsky J. M. Insulin sensitivity: modulation by nutrients and inflammation. The Journal of clinical investigation 118, 2992–3002, 10.1172/JCI34260 (2008). 18769626 PMC2522344

[b4] Johnson A. M. & Olefsky J. M. The origins and drivers of insulin resistance. Cell 152, 673–684, 10.1016/j.cell.2013.01.041 (2013). 23415219

[b5] Shi H. . TLR4 links innate immunity and fatty acid-induced insulin resistance. The Journal of clinical investigation 116, 3015–3025, 10.1172/JCI28898 (2006). 17053832 PMC1616196

[b6] Tsukumo D. M. . Loss-of-function mutation in Toll-like receptor 4 prevents diet-induced obesity and insulin resistance. Diabetes 56, 1986–1998, 10.2337/db06-1595 (2007). 17519423

[b7] Saberi M. . Hematopoietic cell-specific deletion of toll-like receptor 4 ameliorates hepatic and adipose tissue insulin resistance in high-fat-fed mice. Cell metabolism 10, 419–429, 10.1016/j.cmet.2009.09.006 (2009). 19883619 PMC2790319

[b8] Himes R. W. & Smith C. W. Tlr2 is critical for diet-induced metabolic syndrome in a murine model. Faseb J 24, 731–739, 10.1096/Fj.09-141929 (2010). 19841034 PMC2830137

[b9] Uysal K. T., Wiesbrock S. M., Marino M. W. & Hotamisligil G. S. Protection from obesity-induced insulin resistance in mice lacking TNF-alpha function. Nature 389, 610–614 (1997). 9335502 10.1038/39335

[b10] Wen H. T. . Fatty acid-induced NLRP3-ASC inflammasome activation interferes with insulin signaling. Nat Immunol 12, 408–U461, 10.1038/Ni.2022 (2011). 21478880 PMC4090391

[b11] Youm Y. H. . Canonical Nlrp3 Inflammasome Links Systemic Low-Grade Inflammation to Functional Decline in Aging. Cell metabolism 18, 519–532, 10.1016/j.cmet.2013.09.010 (2013). 24093676 PMC4017327

[b12] Spence J., Sadis S., Haas A. L. & Finley D. A Ubiquitin Mutant with Specific Defects in DNA-Repair and Multiubiquitination. Mol Cell Biol 15, 1265–1273 (1995). 7862120 10.1128/mcb.15.3.1265 PMC230349

[b13] Hofmann R. M. & Pickart C. M. Noncanonical MMS2-encoded ubiquitin-conjugating enzyme functions in assembly of novel polyubiquitin chains for DNA repair. Cell 96, 645–653, 10.1016/S0092-8674(00)80575-9 (1999). 10089880

[b14] Laine A. & Ronai Z. Ubiquitin chains in the ladder of MAPK signaling. Science’s STKE: signal transduction knowledge environment 2005, re5, 10.1126/stke.2812005re5 (2005). 15855411

[b15] Chen Z. J., Bhoj V. & Seth R. B. Ubiquitin, TAK1 and IKK: is there a connection? Cell Death Differ 13, 687–692, 10.1038/sj.cdd.4401869 (2006). 16485032

[b16] Deng L. . Activation of the IkappaB kinase complex by TRAF6 requires a dimeric ubiquitin-conjugating enzyme complex and a unique polyubiquitin chain. Cell 103, 351–361 (2000). 11057907 10.1016/s0092-8674(00)00126-4

[b17] Wang C. . TAK1 is a ubiquitin-dependent kinase of MKK and IKK. Nature 412, 346–351, 10.1038/35085597 (2001). 11460167

[b18] Habelhah H. . Ubiquitination and translocation of TRAF2 is required for activation of JNK but not of p38 or NF-kappa B. Embo J 23, 322–332, 10.1038/sj.emboj.7600044 (2004). 14713952 PMC1271753

[b19] Baud V. & Karin M. Signal transduction by tumor necrosis factor and its relatives. Trends in cell biology 11, 372–377 (2001). 11514191 10.1016/s0962-8924(01)02064-5

[b20] Aggarwal B. B. Signalling pathways of the TNF superfamily: a double-edged sword. Nature reviews. Immunology 3, 745–756, 10.1038/nri1184 (2003). 12949498

[b21] Akira S. & Takeda K. Toll-like receptor signalling. Nature Reviews Immunology 4, 499–511, 10.1038/Nri1391 (2004). 15229469

[b22] Fukushima T. . Ubiquitin-conjugating enzyme Ubc13 is a critical component of TNF receptor-associated factor (TRAF)-mediated inflammatory responses. P Natl Acad Sci USA 104, 6371–6376, 10.1073/pnas.0700548104 (2007). PMC1851032 17404240

[b23] Chiang S. H. . The protein kinase IKKepsilon regulates energy balance in obese mice. Cell 138, 961–975, 10.1016/j.cell.2009.06.046 (2009). 19737522 PMC2756060

[b24] Reilly S. M. . An inhibitor of the protein kinases TBK1 and IKK-varepsilon improves obesity-related metabolic dysfunctions in mice. Nature medicine 19, 313–321, 10.1038/nm.3082 (2013). PMC3594079 23396211

[b25] Ozcan U. . Endoplasmic reticulum stress links obesity, insulin action, and type 2 diabetes. Science (New York, N.Y.) 306, 457–461, 10.1126/science.1103160 (2004). 15486293

[b26] Tcherpakov M. . Regulation of endoplasmic reticulum-associated degradation by RNF5-dependent ubiquitination of JNK-associated membrane protein (JAMP). J Biol Chem 284, 12099–12109, 10.1074/jbc.M808222200 (2009). 19269966 PMC2673279

[b27] Hotamisligil G. S. Endoplasmic reticulum stress and the inflammatory basis of metabolic disease. Cell 140, 900–917, 10.1016/j.cell.2010.02.034 (2010). 20303879 PMC2887297

[b28] Ozcan U. . Chemical chaperones reduce ER stress and restore glucose homeostasis in a mouse model of type 2 diabetes. Science (New York, N.Y.) 313, 1137–1140, 10.1126/science.1128294 (2006). PMC4741373 16931765

[b29] Zhang H. . STAT3 restrains RANK- and TLR4-mediated signalling by suppressing expression of the E2 ubiquitin-conjugating enzyme Ubc13. Nature communications 5, 5798, 10.1038/ncomms6798 (2014). PMC4270087 25503582

[b30] Dziennis S. . Role of signal transducer and activator of transcription-3 in estradiol-mediated neuroprotection. The Journal of neuroscience: the official journal of the Society for Neuroscience 27, 7268–7274, 10.1523/jneurosci.1558-07.2007 (2007). 17611279 PMC2570353

[b31] Madiraju C. . TR-FRET-based high-throughput screening assay for identification of UBC13 inhibitors. Journal of biomolecular screening 17, 163–176, 10.1177/1087057111423417 (2012). 22034497 PMC4172584

[b32] Yamamoto M. . Key function for the Ubc13 E2 ubiquitin-conjugating enzyme in immune receptor signaling. Nat Immunol 7, 962–970, 10.1038/ni1367 (2006). 16862162

[b33] Yamamoto M. . Cutting Edge: Pivotal function of Ubc13 in thymocyte TCR signaling. Journal of immunology 177, 7520–7524 (2006). 10.4049/jimmunol.177.11.7520 17114420

[b34] Sayama K. . E2 Polyubiquitin-conjugating Enzyme Ubc13 in Keratinocytes Is Essential for Epidermal Integrity. J Biol Chem 285, 30042–30049, 10.1074/jbc.M110.106484 (2010). 20663875 PMC2943323

[b35] Chang J. H. . Ubc13 maintains the suppressive function of regulatory T cells and prevents their conversion into effector-like T cells. Nat Immunol 13, 481–U484, 10.1038/Ni.2267 (2012). 22484734 PMC3361639

[b36] Naitoh R. . Inhibition of GIP signaling modulates adiponectin levels under high-fat diet in mice. Biochem Bioph Res Co 376, 21–25, 10.1016/j.bbrc.2008.08.052 (2008). 18723001

[b37] Lusk G. Animal calorimetry. Twenty-fourth paper. Analysis of the oxidation of mixtures of carbohydrate and fat. A correction. J Biol Chem 59, 41–42 (1924).

[b38] Frayn K. N. Calculation of substrate oxidation rates *in vivo* from gaseous exchange. Journal of applied physiology: respiratory, environmental and exercise physiology 55, 628–634 (1983). 10.1152/jappl.1983.55.2.628 6618956

[b39] Nasteska D. . Chronic Reduction of GIP Secretion Alleviates Obesity and Insulin Resistance Under High-Fat Diet Conditions. Diabetes 63, 2332–2343, 10.2337/Db13-1563 (2014). 24584548

[b40] Joo E. . Enteral supplement enriched with glutamine, fiber, and oligosaccharide attenuates experimental colitis in mice. Nutrition 29, 549–555, 10.1016/j.nut.2012.09.007 (2013). 23274091

