# Supplementary material for: Ubc13 haploinsufficiency protects against age-related insulin resistance and high-fat diet-induced obesity
Source: Sci Rep. 2016 Oct 31;6:35983. doi: 10.1038/srep35983 (PMC5086849; doi:10.1038/srep35983)
Supplement: Supplementary Information [file srep35983-s1.pdf]

# Supplementary information

## Ubc13 haploinsufficiency protects against age-related insulin resistance and high-fat diet-induced obesity

Erina Joo, Toru Fukushima, Norio Harada, John C. Reed, Shu-ichi Matsuzawa, and Nobuya Inagaki

**Fig. S1. Male *ubc13*$^{+/-}$ mice tend to display decreased weight gain on HFD.** Body weights of male WT versus *ubc13*$^{+/-}$ mice on ND or HFD (n = 6). HFD was started at 4 weeks of age.

**Fig. S2. There is no significant difference in food intake and energy expenditure between WT and *ubc13*$^{+/-}$ mice on ND.** (*A* and *B*) Food intake (*A*) and energy expenditure (*B*) were measured for female WT and *ubc13*$^{+/-}$ mice on a ND (n = 4).

**Fig. S3. Effect of Ubc13 haploinsufficiency on insulin sensitivity in male mice.** (*A, B,* and *C*) Blood glucose (*A*) and serum insulin (*B*) levels during OGTT and blood glucose (*C*) levels during ITT were measured for male WT and *ubc13*$^{+/-}$ mice at 18 weeks of age on ND (n = 5 to 6). * $p < 0.05$ versus WT mice. (*D, E,* and *F*) Blood glucose (*D*) and serum insulin (*E*) levels during OGTT and blood glucose (*F*) levels during ITT were measured for male WT and *ubc13*$^{+/-}$ mice after 14 weeks of HFD (n = 5 to 6). * $p < 0.05$ versus WT mice. HFD was started at 4 weeks of age.

**Fig. S4. There is no significant difference in liver and VAT weights between WT and *ubc13*$^{+/-}$ mice on ND.** (*A* and *B*) Liver and VAT weights of female WT and *ubc13*$^{+/-}$ mice were measured for female WT and *ubc13*$^{+/-}$ mice after 14 weeks of ND (n = 6).

**Fig. S5. Female *ubc13*$^{+/-}$ mice are protected against HFD-induced skeletal mucle insulin resistance.** Quadriceps muscle lysates from female WT and *ubc13*$^{+/-}$ mice on HFD were analyzed by immunoblotting using antibodies for p-Akt (Ser473), total Akt, and Ubc13. The membrane was reprobed with anti-HSP90 antibody as a control. The intensities of phosphorylated-Akt after insulin injection were normalized to total Akt protein levels. (B) Representative photographs of H&E staining of muscle from female WT and *ubc13*$^{+/-}$ mice on

HFD.

**Fig. S6. There is no significant difference in the *TNF-$\alpha$, IL-6, IL-1$\beta$, IKK$\varepsilon$,* and *F4/80* mRNA levels in VAT between male WT and *ubc13*$^{+/-}$ mice on HFD.** (*A*) qRT-PCR analysis of the expression of the genes encoding *TNF$\alpha$, IL-6, IL-1$\beta$,* and *IKK$\varepsilon$* in VAT of male WT and *ubc13*$^{+/-}$ mice on HFD (n = 4 to 6). (*B*) qRT-PCR analysis of the expression of the gene encoding *F4/80* in VAT of male WT and *ubc13*$^{+/-}$ mice on HFD (n = 4 to 6).

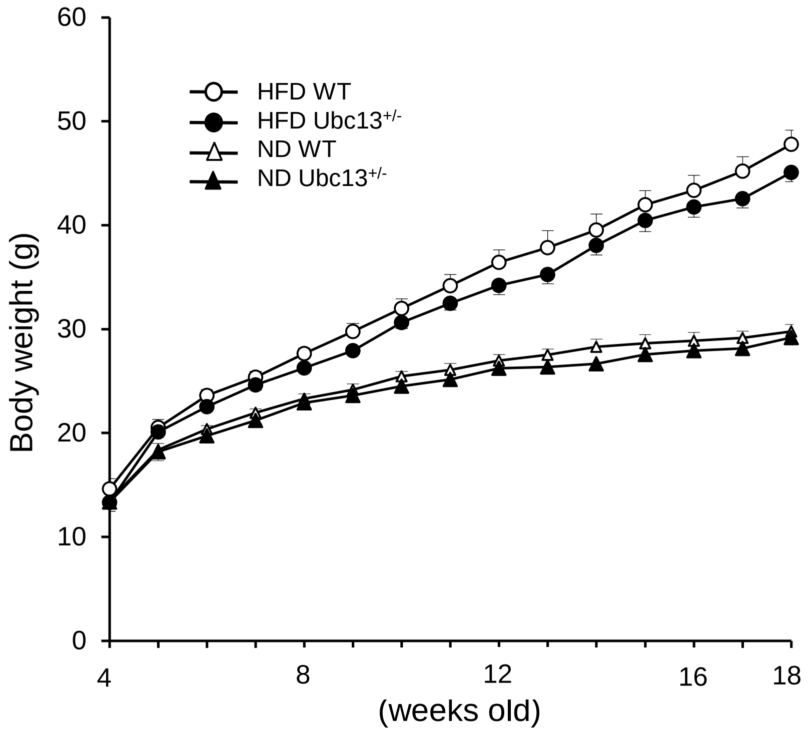
HFD WT
HFD Ubc13+/-
ND WT
ND Ubc13+/-
60
50
40
30
20
10
0
Body weight (g)
4
8
12
16
18
(weeks old)

Figure S1

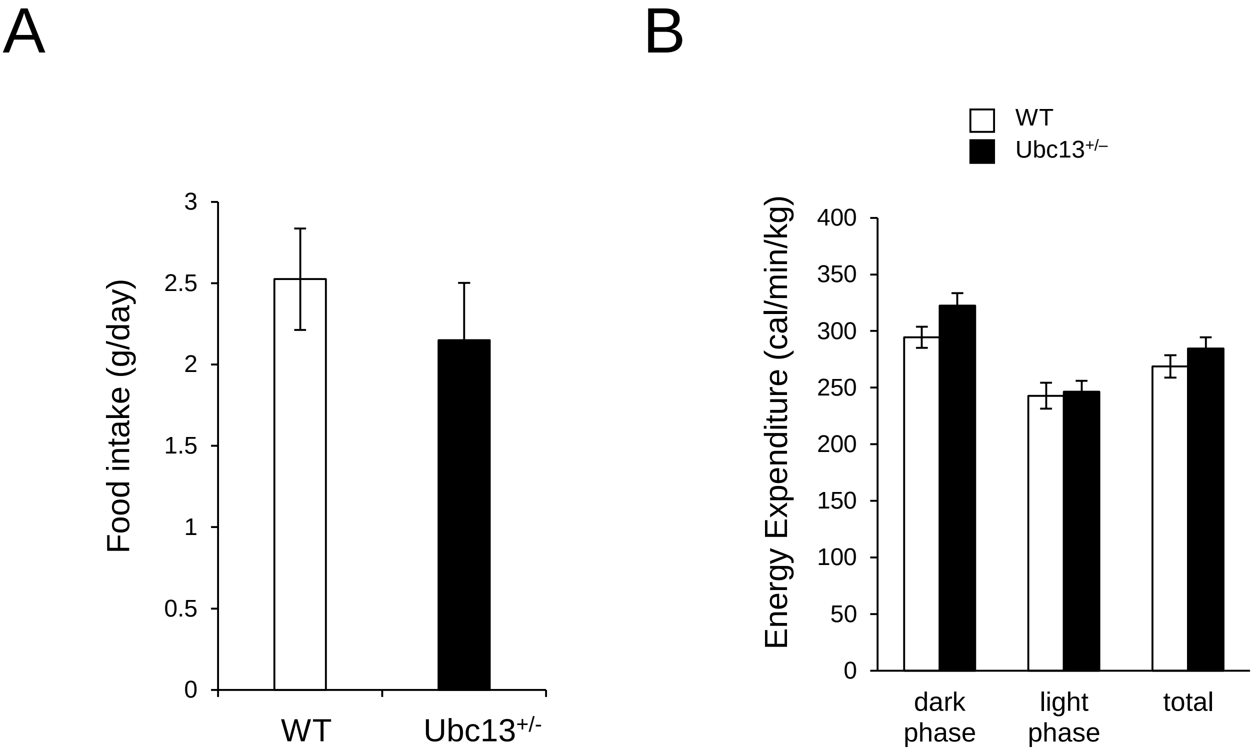
A
B
Food intake (g/day)
3
2.5
2
1.5
1
0.5
0
WT
Ubc13+/-
WT
Ubc13+/–
Energy Expenditure (cal/min/kg)
400
350
300
250
200
150
100
50
0
dark phase
light phase
total

Figure S2

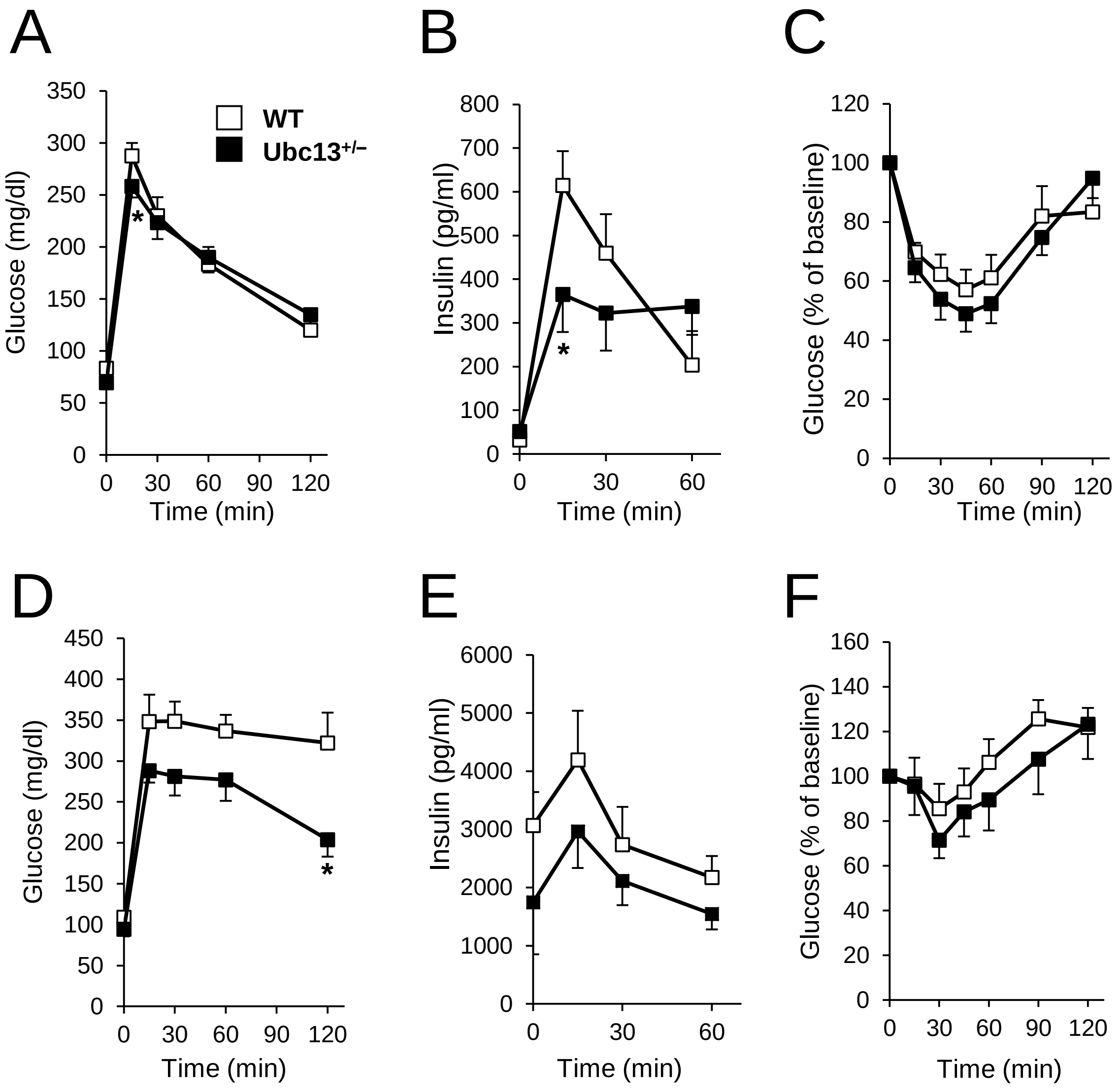
A
B
C
D
E
F
WT
Ubc13+/–
Glucose (mg/dl)
Insulin (pg/ml)
Glucose (% of baseline)
Time (min)
*

Figure S3

A B

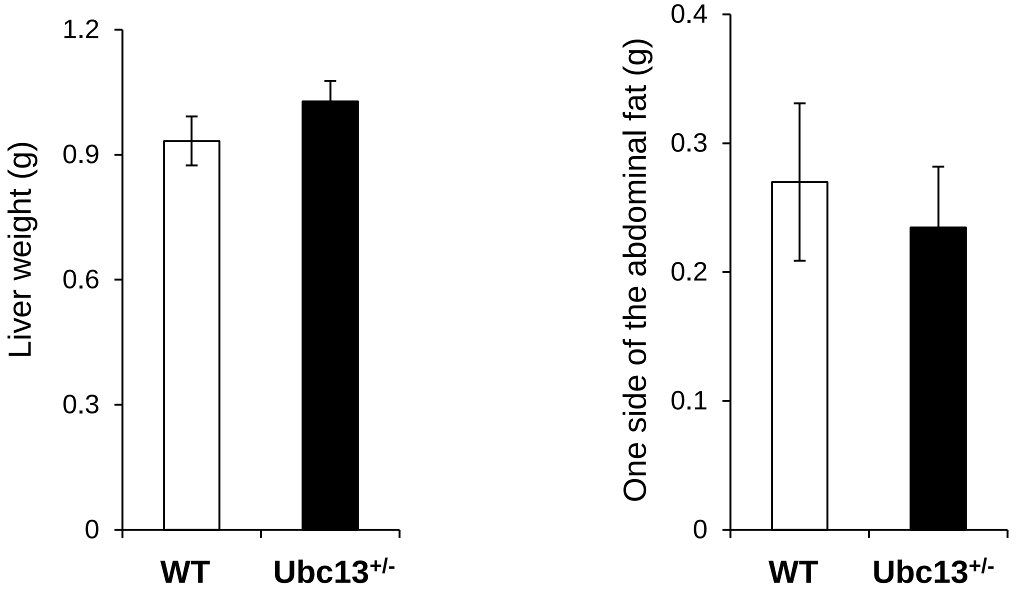
1.2
0.9
0.6
0.3
0
Liver weight (g)
WT
Ubc13+/-
0.4
0.3
0.2
0.1
0
One side of the abdominal fat (g)
WT
Ubc13+/-

Figure S4

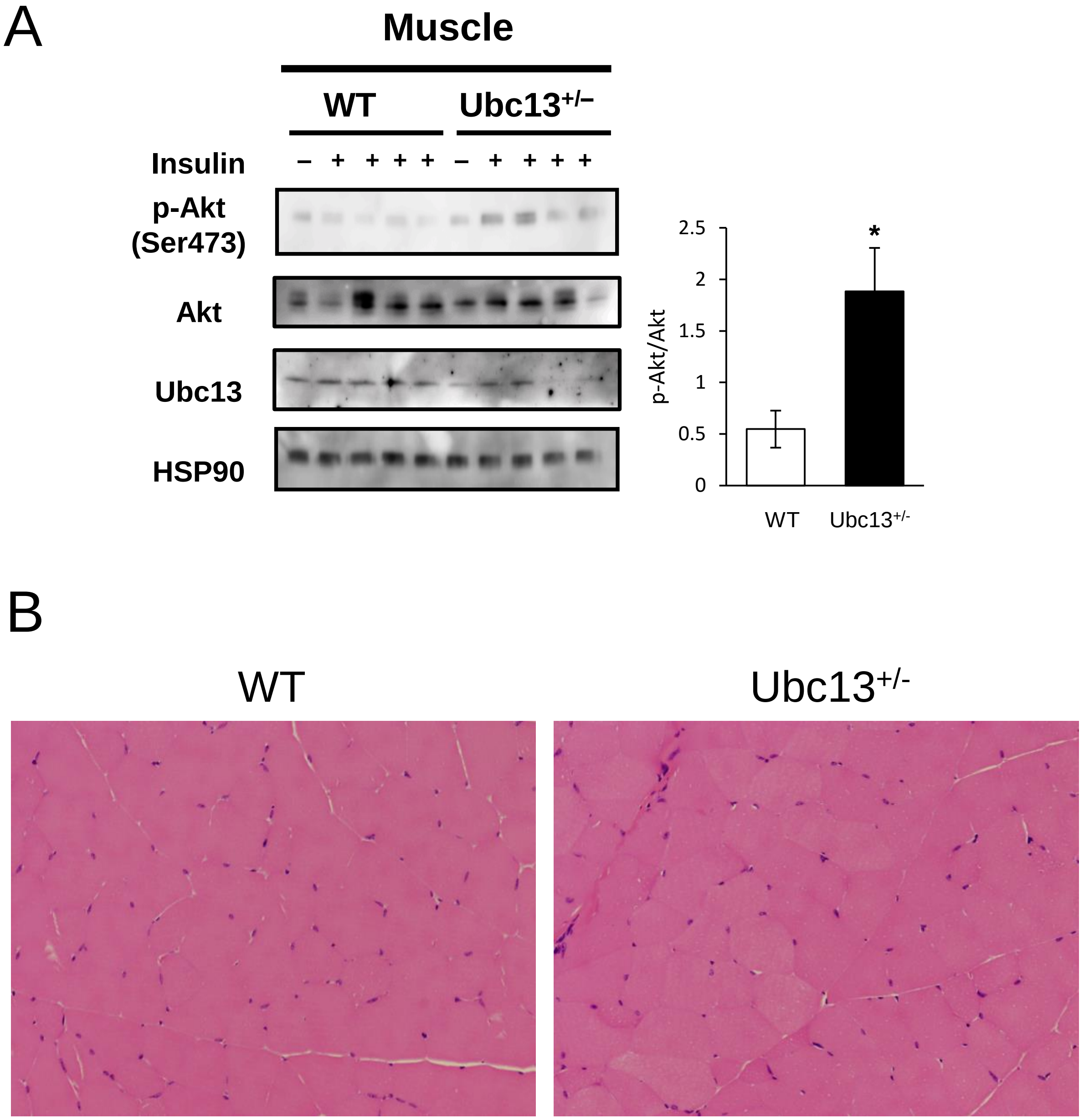
A
Muscle
WT
Ubc13+/–
Insulin
– + + + + – + + + +
p-Akt
(Ser473)
Akt
Ubc13
HSP90
p-Akt/Akt
2.5
2
1.5
1
0.5
0
*
WT
Ubc13+/-
B
WT
Ubc13+/-

Figure S5

A

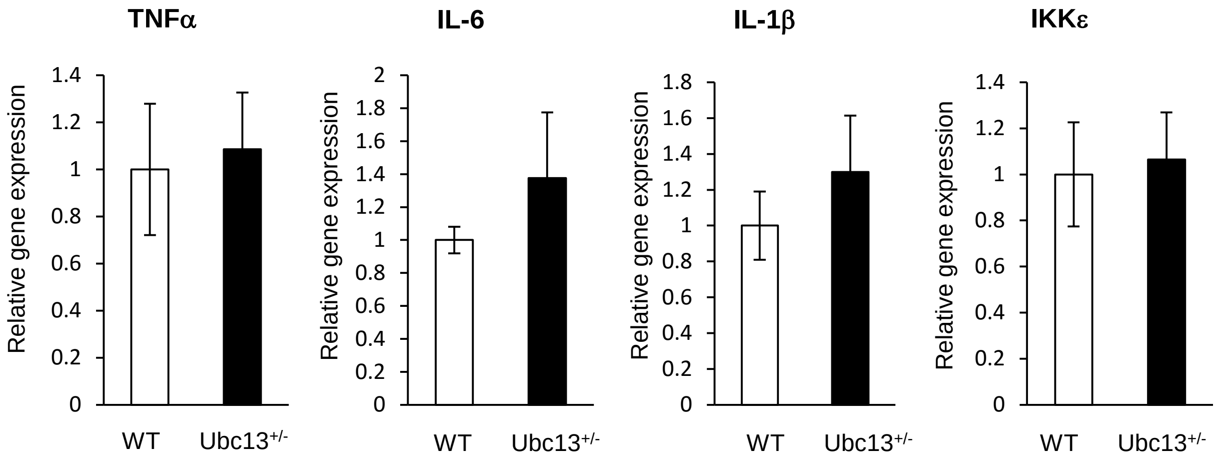
TNFα
IL-6
IL-1β
IKKε
Relative gene expression
WT
Ubc13+/-

B

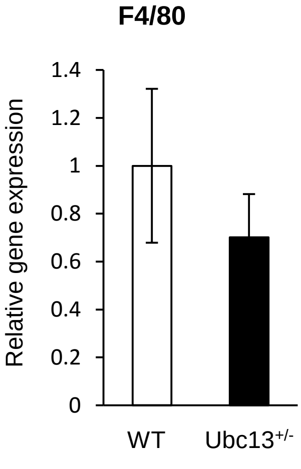
F4/80
Relative gene expression
WT
Ubc13+/-

Figure S6
